# Positive versus negative pressure during removal of endotracheal-tube on prevention of post-extubation atelectasis in ventilated neonates: A randomized controlled trial

**DOI:** 10.1016/j.amsu.2022.103573

**Published:** 2022-04-04

**Authors:** Roya Farhadi, Maryam Nakhshab, Atefeh Hojjati, Mohammad Khademloo

**Affiliations:** a Pediatrics Department, School of Medicine, Mazandaran University of Medical Sciences, Sari, Iran; b Department of Community Medicine, School of Medicine, Mazandaran University of Medical Sciences, Sari, Iran

**Keywords:** Tracheal extubation, Neonate, Extubation methods, Post-extubation atelectasis, PEA, Post extubation atelectasis, PEEP, Positive End Expiratory Pressure, CPAP, Continuous Positive Airway Pressure, ETT, Endo Tracheal Tube

## Abstract

**Background:**

Post-extubation-atelectasis (PEA) is a common problem after the removal of an endotracheal tube in neonates which increases the rate of extubation failure. Different techniques have been introduced for the prevention of PEA. One technique is the removal of the endotracheal tube by negative or positive gradients of pressure. No RCT has yet been done to compare the use of these two methods in neonates. So this study aimed to compare the role of positive and negative pressure during extubation of neonates on the prevention of PEA.

**Materials and methods:**

We enrolled 100 newborns in this RCT that required at least 24 h of mechanical ventilation. The endotracheal tube in one group was removed by a T-Piece resuscitator at a PEEP level of 5 CmH2o while in another group extubation was done applying suction pressure of 100 mmHg by random selection. Prevalence of PEA in CXRs after extubation was compared between the two groups.

**Results:**

The prevalence of PEA in the extubation of the positive pressure group (24%) was significantly lower than that of the negative pressure group (46%) (p = 0.024). Extubation failure was found to be lower in the positive pressure group (6% versus 20% P = 0.037). No significant difference was observed between the two groups in the prevalence of apnea, pneumothorax, and death at 3 days after extubation.

**Conclusion:**

The use of positive pressure during removal of the endotracheal tube in newborn infants reduced the rate of PEA compared with the negative pressure so extubation by a positive pressure is recommended in neonates.

## Highlights

• Post extubation atelectasis (PEA) is a common problem after the removal of an endotracheal tube in neonates.
• This study compares two different extubation methods for the prevention of PEA in neonates.
• The prevalence of PEA in the extubation with positive pressure method was significantly lower than the negative pressure.
• Extubation by a positive pressure method is recommended in neonates.

## 1 Introduction

Maintaining an adequate gas exchange is one of the main goals in mechanical ventilation of neonates suffering from respiratory failure. Meanwhile, another important aim is early extubation for minimizing the risk of prolonged mechanical ventilation such as poor neurologic outcome and bronchopulmonary dysplasia [[1], [2], [3], [4]]. However, extubation failure is a major problem after extubation in neonatal intensive care units that increases comorbidities and mortality [5]. A rate of 30–50% has been reported in studies, which could even go higher at lower gestational age [6,7]. Reintubation is needed in up to 40% of extremely low birth weight infants [8]. Furthermore, reintubation leads to traumatic injury to the upper airway, infection, and pulmonary atelectasis [5,9].In addition, mechanical ventilation is still considered an important predisposing factor for the development of chronic lung disease and no significant decrease has been reported in the rate of this complication [[10], [11], [12]].

Post extubation atelectasis (PEA) is one of the causes of extubation failure [13]. PEA leads to the collapse of the lung in ventilated neonates and increases the hospitalization time of neonates in neonatal intensive care units [14,15]. Prematurity, prolonged and recurrent intubation, low birth weight, patency of ductus arteriosus, nasotracheal intubation are known as predisposing factors of PEA and the right lung is the most common site of PEA [5]. Small airway size and muscle weakness in neonates contribute to obstructing effects of accumulating airway secretions which is the primary cause of atelectasis [16].

In some studies, different methods such as the implementation of chest physiotherapy after extubation, use of Continuous Positive Airway Pressure (CPAP) after removal of an endotracheal tube (ETT) in premature infants, and orotracheal insertion instead of nasotracheal intubation have been recommended for prevention of PEA [16,17].

Direct removal of the ETT during extubation could lead to the collapse of neonatal airways due to sudden deflation of ventilated lungs. So, the removal of an ETT is an important step in a ventilated neonate's course [18]. The benefits of extubation with positive pressure have been emphasized by many authors; however, some clinicians still use negative pressure during this procedure [19]. To the best of our knowledge, and according to the previous evidence, no randomized controlled trial has been done yet regarding the advantage of these two methods to prevent PEA [20]. Indeed some studies about various techniques of extubation including suctioning the trachea, adjusting the positive end expiratory pressure (PEEP) setting on the ventilator, or compressing the self-inflating bag just before the removal of the endotracheal tube has been carried out in adult patients [21], but in the neonatal group, no evidence exists about the best techniques for extubation [22,23]. On the other hand, applying PEEP during the removal of the ETT can be protective against the aspiration of secretions [23]. So, a randomized controlled trial was conducted to investigate whether applying positive pressure during the removal of the ETT is capable of reducing the rate of PEA compared with negative pressure. Meanwhile, this study aimed at comparing the rate of extubation failure between these two techniques.

## 2 Material and methods

This study is an interventional randomized controlled trial (RCT) that was conducted in a level III neonatal intensive care unit in Boo-Ali Sina Hospital in Sari (north of Iran) between January 2016 and May 2020.

### 2.1 Ethical consideration

The approval of the Ethics Committee for Research was obtained from Mazandaran University of Medical Sciences. The study has been reported in line with the (CONSORT) criteria (http://www.journal-surgery.net/article/S1743-9191%2811%2900565-6/fulltext) [24]. Informed consent was obtained from patients’ guardians. The trial was registered in the Iranian Registry of Clinical Trials (IRCT201112092801N2) which is a Primary Registry in the WHO Registry Network (https://www.irct.ir/trial/2645).

### 2.2 Inclusion and exclusion criteria

All neonates who were under mechanical ventilation via an orotracheal tube for at least 24 h and ready for first extubation were eligible. Neonates with the following conditions were excluded from the study: bronchopulmonary dysplasia, major congenital anomalies, congenital neuromuscular disorders, hemodynamically significant patent ductus arteriosus in echocardiography, intraventricular hemorrhage grade III or IV, nasotracheal intubation, and meconium aspiration or bacterial pneumonia.

### 2.3 Sample size

We estimated a sample size of 52 in each group based on about 25% differences in the incidence of PEA between two groups in a pilot study with an α error of 5% and 80% power.

### 2.4 Sampling technique and procedures

A chest x-ray before extubation was obtained for all eligible neonates and the correct position of the endotracheal tube was checked by a neonatologist. Feeding was withheld for 4 h before and at least 24 h after extubation. The stomach was aspirated before the removal of the ETT. Dexamethasone was used if the neonate required more than seven days of mechanical ventilation. Intravenous caffeine citrate was started at pre-extubation in neonates <37 weeks of gestation and continued during the next week. All sedative or muscle paralyzing drugs were discontinued at least 24 h before extubation. The suction of the endotracheal tube and oropharynx was done 5 min before the procedure, and we ensured that vital signs have been returned to the baseline before extubation. The decision to extubate the patients was determined by a neonatologist according to the following criteria: a) infants being clinically and hemodynamically stable. b) normal lab data: acceptable blood gas, hematocrit >30%, normal blood sugar and electrolytes c) ventilator settings: PIP 12–14 CmH2O, PEEP 4 CmH2O, FIo2 < 40%, Rate 15–20/min) if neonates could tolerate pressure support mode of ventilation for at least 24 h and be stable during this time. Afterward, informed consent was obtained from the parents. At the time of extubation, in a parallel-group design, eligible infants were randomly assigned to receive either a positive or a negative pressure during the removal of the ETT. Randomization was designed as concurrent active control by the computer software program that generated the random sequence in sealed opaque envelopes, which were drawn before extubation by a pediatric resident in the team who was responsible for removing the ETT. One group, after removing ETT tapes, was extubated by a negative pressure adjusted to 100 mmHg via a suction catheter entered into the endotracheal tube during its removal. In another group, the endotracheal tube was connected to an infant T-Piece resuscitator (Neopuff, Fisher&Paykel New Zealand) during the removal of the ETT which PEEP level in the T-Piece resuscitator was adjusted by turning the PEEP cap in 5 CmH2O on a flow rate of 8 L per minute.

Neonates in each group were stratified into two blocks by their birth weight including: <2000 g and ≥2000 g. Neonates with a birth weight less than 2000 g were categorized as <1000 g and 1000–2000 g. Post-extubation nasal CPAP was applied in all neonates with a pressure level of 5 CmH2O to keep the arterial oxygen saturation between 90 and 95%. Post extubation clinical and nursing care such as the suction of airway secretions were done equally for the two groups according to our center's protocol. All patients were followed under continuous monitoring of vital signs and oxygen saturation until clinical stabilization for at least 72 h. A Chest x-ray was taken 24 h after extubation.

### 2.5 Primary and secondary outcomes

PEA as a primary outcome of our trial was defined as an opacification on the chest x-ray with loss of lung volume and was diagnosed by another neonatologist in the team and confirmed by a radiologist. It was diagnosed according to evidence of a new post-extubation pulmonary collapse in parts of the lungs compared to a pre-extubation chest x-ray. Both the radiologist and neonatologist were unaware of the study group assignment. In case of disagreement between the radiologist and neonatologist in diagnosis, the patient was excluded from the study.

Patients’ information was recorded including age, birth weight, sex, gestational age, primary diagnosis, duration of ventilator therapy, and reintubation (if needed) during the admission period. Extubation failure was defined as the need for reintubation 48 h after the removal of the endotracheal tube which was indicated by the presence of respiratory acidosis, (pH.<7.20 and PCo2> 60 mmHg), increasing oxygen requirement (PO2<50 mmHg or Spo 2< 90% with FIO2≥70%) and repeated episodes of apnea [25,26]. In addition to the evidence of PEA as a primary outcome, an extubation failure and other outcomes including the occurrence of apnea, pneumothorax, or death within 72 h after extubation has been documented as the secondary outcome. The patient was excluded from the final analysis of the incidence of PEA if death occurred within 24 h after extubation.

### 2.6 Statistical analysis

Continuous variables between groups were compared using Student's t-test or Mann-Whitney *U* test. Pearson's chi-square test was used for categorical variables. Data were analyzed by SPSS V.18. Statistical significance was determined by a P-value <0.05.

## 3 Results

### 3.1 Demographic characteristics of study patients

**Fig. 1:**
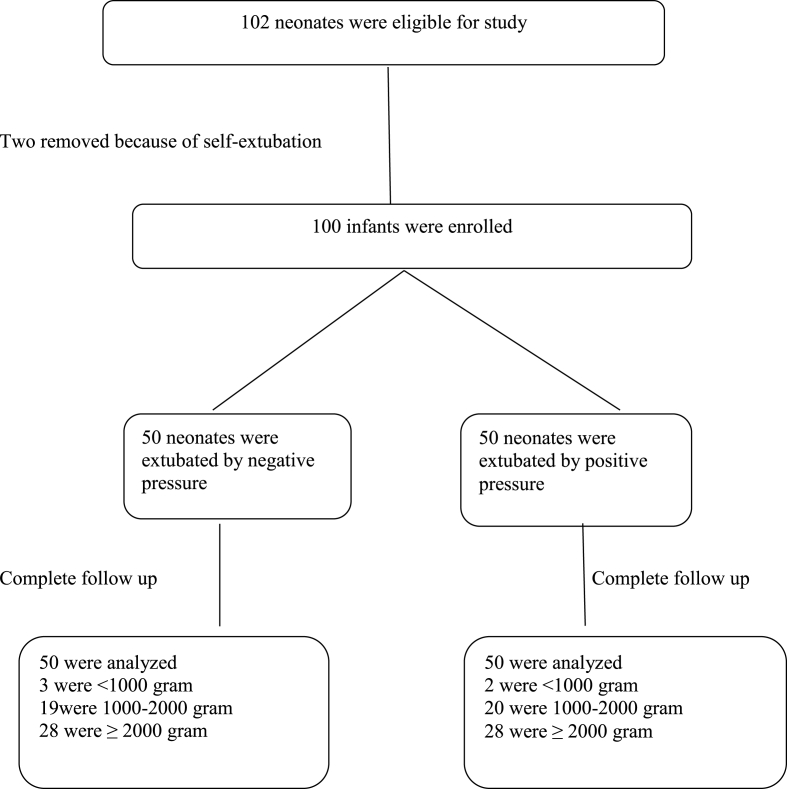
Flow of randomization and enrollment process of study.

In this RCT, 102 neonates were eligible for the study. Two neonates, one from each group, were withdrawn from the study due to self-extubation at the beginning of the study. Finally, 100 infants were enrolled. Infants were randomized to receive either a positive (n = 50) or a negative (n = 50) pressure during the removal of the ETT as a two-weight blockage (Fig. 1).

Demographic characteristics including gestational age, birth weight, age of the neonate at the time of extubation, sex and duration of ventilator therapy and use of medication before extubation were similar between the two groups (Table 1).

**Table 1:** Demographics variables of neonates between two groups.

Variable	Positive pressure	Negative pressure	P-value
Age of extubation (day) (mean ± SD)			
		
	13.04 ± 0.8	13.6 ± 10.8	0.71
Sex (N, %)			
Male	30(60%)	32(64%)	
Female	20(40%)	18(36%)	0.68
Birth weight(gram) (mean ± SD)	2388 ± 904.7	2307 ± 848.4	0.64
Duration of ventilator therapy(day) (mean ± SD)	7.6 ± 5.7	8.8 ± 7.4	0.65
Gestational age(week) (mean ± SD)	34.4 ± 3.9	34.06 ± 3.6	0.6
Use of Dexamethasone (N, %)	13(26%)	12(24%)	0.69
Use of caffeine citrate (N, %)	33(66%)	35(70%)	0.64

### 3.2 Comparison of outcomes between two groups

In the positive pressure group, 32 neonates (64%) and in the negative pressure group 31 neonates (62%) suffered from respiratory distress syndrome (RDS). Neonatal seizure, Meningitis, surgical diseases including intestinal atresia and malrotation, necrotizing enterocolitis (NEC), and apnea of prematurity were other causes of ventilator therapy. There was no significant difference between the two groups regarding the underlying diseases (P = 0.12).

PEA was documented in 23 (46%) of the neonates extubated with negative pressure, and in 12(24%) infants being extubated with positive pressure. These rates indicated a significant difference between the two groups (P = 0.024). Analysis based on birth weight also showed a significant difference in the block of patients weighing <2000 g (range 800–1950 g). This difference was also significant in patients with a weight of 1000–2000 g.

The most frequent site of PEA in this study was the right upper lobe of the lung (38 and 36 cases in positive and negative pressure groups respectively). Ten cases from the positive and 8 cases from the negative group had right middle lobe atelectasis. Right lower lobe atelectasis was observed in 7 patients and one case from the positive group had the collapse of both lungs.

The rate of extubation failure was 20% (n = 10) in the negative pressure group while it was found in 6% (n = 3) in the positive pressure group (P = 0.037). Although according to the birth weight categorization the rate of extubation failure was greater in (1000–2000 g and ≥2000 g) neonates who were extubated by negative pressure, the statistical analysis showed no significant difference. Other outcomes including post-extubation apnea, pneumothorax, and death within three days after extubation were not significantly different between the two groups (Table 2).

**Table 2:** Comparison of outcomes between two groups.

Outcome	Positive pressure	Negative pressure	P-value
aPEA total (N, %)	12(24%)	23(46%)	0.024
<2000 g	5(22.7%)	10(45.4%)	0.048
<1000 g	1(50%)	0(0)	0.4
1000–2000 g	4(20%)	10(52.6%)	0.048
≥2000 g	7(25%)	13(46.4%)	0.166
bEF total (N, %)	3(6%)	10(20%)	0.037
<2000 g	2(9.1%)	4(18.8%)	0.34
<1000 g	1(50%)	1(33.3%)	0.7
1000–2000 g	1(5%)	3(15.8%)	0.34
≥2000 g	1(3.6%)	6(21.4%)	0.1
Post extubation apnea (N, %)	2(4%)	2(4%)	1
Post extubation pneumothorax (N, %)	1(2%)	0(0)	0.5

a Post Extubation Atelectasis.

b Extubation Failure.

## 4 Discussion

PEA is one of the causes of extubation failure in neonates that led to reintubation which is associated with tracheal damage. Previous studies state that about 10–50% of newborns develop atelectasis after extubation [27]. This is the first trial sought to determine the best technique of extubation in neonates. The findings of our study revealed a significantly lower incidence of PEA in the removal of ETT by a positive pressure compared to the use of the negative pressure during extubation. On the other hand, this trial showed a higher incidence of extubation failure in the negative pressure group. Hiremath et al. in a prospective observational study found that PEA was one of the common causes of extubation failure [13]. Therefore, we suggest that the removal of ETT by applying positive pressure could decrease the incidence of extubation failure due to reducing PEA. So, it seems that the extubation technique has an important role in the failure of extubation in neonates.

The effect of positive pressure during the removal of ETT had been studied by Rassam et al. The authors concluded that the positive pressure generated before removing the tracheal tube helps to prevent secretions from tracking into the larynx and inducing laryngeal spasm. The positive pressure in their study was supplied with a simple basic maneuver by squeezing the reservoir bag. They believed that the use of this technique and other new methods should be considered evidence-based and well researched because this study was a postal survey in which anesthetists replied to a questionnaire about the management of the airway during the extubation period [28]. This study included only adult patients and after anesthesia while our study was designed as an RCT in the neonatal period. On the other hand, we used a T-Piece resuscitator with a specified PEEP level.

Gentile et al. in a review article about recent advances in the treatment of ventilator-associated pneumonia reported different methods to clear the subglottic secretions. They described applying a positive pressure gradient during the time of extubation. Their hypothesis asserts that secretions would be removed from the subglottic space by the escaping gas in the oropharyngeal space where they can be suctioned away. They recommended further studies on this technique [29]. Their review included only adult patients. Siobal et al. described the use of a constant flow inflating resuscitating bag for purging of the subglottic space during the extubation process. Despite the description of this technique in respiratory care textbooks, only low-level evidence-based study exists to support the routine use of this method and the knowledge about the potential benefit of this simple technique is still low [22]. Hodd et al. in two studies reported that the use of a positive end expiratory pressure either by compressing a self-inflating bag or adjusting a PEEP level on the ventilator during the extubation process minimized subglottic secretions aspiration but none of these studies were done in the neonatal period [23,30].

To the best of our knowledge, our study is the first study in this regard in the neonatal group. It seems the use of PEEP similar to adult patients reduces the risk of PEA and extubation failure. Based on previous studies that examined risk factors of PEA and examined the role of positive pressure before removal of the endotracheal tube it may be because of decreasing aspiration of secretions, increasing the functional residual capacity, and the prevention of small airway collapse during extubation [28]. In our study, no correlation was found between post-extubation apnea, pneumothorax, and the technique of extubation. Although the overall incidence of PEA was lower in the group receiving positive pressure, no significant differences were observed in the block of neonates equal to or above 2000 g between the two groups.

### 4.1 Strength and limitations of the study

The strength of our study is that to date no study has investigated the technique of endotracheal tube removal in neonates, and this study is the first study with a reasonable sample size to address the role of pressure gradients in the success of extubation in the neonatal age group. However, our study has some limitations. First, we studied the neonates of a single-center, so it is recommended to conduct a multicenter study to make a better conclusion. Second, the number of our babies has been low in some weight categories. This may be because of the lack of the number of neonates in each block concerning our RCT randomization and enrollment. So further research based on the use of this technique in different weight categories of neonates with larger sample sizes and longer follow-up is required.

## 5 Conclusion

A positive pressure during extubation reduces the risk of PEA and extubation failure and this technique is recommended during the removal of the endotracheal tube in neonates.

## Ethical approval

Ethical approval was obtained for this study from the research ethics committee of Mazandaran University of Medical Sciences (No. 90–113). The trial was registered in the Iranian Registry of Clinical Trials (IRCT201112092801N2) which is a Primary Registry in the WHO Registry Network.

## Sources of funding

This project resulted from the residency thesis of Atefeh Hojjati and was supported by grant (90-113) from 10.13039/501100004160Mazandaran University of Medical Sciences.

## Author contributions

Roya Farhadi is responsible for designing and conducting the study and developed the first draft manuscript. Maryam Nakhshab supervised the study and edited the manuscript. Atefeh Hojjati performed extubation of patients and clinical data collection. Mohammad Khademloo is responsible for data interpretation and analysis. All authors agreed on the final submitted version of the manuscript.

## Trail registry number

1. Name of the registry: Iranian Registry of Clinical Trials
2. Unique Identifying number or registration ID: IRCT201112092801N2
3. Hyperlink to your specific registration (must be publicly accessible and will be checked): https://www.irct.ir/trial/2645

## Guarantor

Dr. Roya Farhadi.

## Consent

The approval of the Ethics Committee for Research was obtained from Mazandaran University of Medical Sciences. (No. 90–113). *Informed consent* was *obtained from patients’ guardians.*

## Availability of data and materials

All data and material collected during this study are available from the corresponding author upon reasonable request.

## Provenance and peer review

Not commissioned, externally peer-reviewed.

## Declaration of competing interest

The authors declare that they have no competing interests.
